# Suppressive Effect of the α-Amylase Inhibitor Albumin from Buckwheat (*Fagopyrum esculentum* Moench) on Postprandial Hyperglycaemia

**DOI:** 10.3390/nu10101503

**Published:** 2018-10-15

**Authors:** Kazumi Ninomiya, Shigenobu Ina, Aya Hamada, Yusuke Yamaguchi, Makoto Akao, Fumie Shinmachi, Hitoshi Kumagai, Hitomi Kumagai

**Affiliations:** 1 Department of Food Science and Nutrition, Kyoritsu Women’s University, 2-2-1 Hitotsubashi, Chiyoda-ku, Tokyo 101-8347, Japan; kninomiya@kyoritsu-wu.ac.jp (K.N.); kumagai@kyoritsu-wu.ac.jp (H.K.); 2 College of Bioresource Sciences, Nihon University, 1866 Kameino, Fujisawa-shi, Kanagawa 252-0880, Japan; carlosshige@yahoo.co.jp (S.I.); hama-aya527@snow.ocn.ne.jp (A.H.); yamaguchi.yusuke@nihon-u.ac.jp (Y.Y.); makao01@brs.nihon-u.ac.jp (M.A.); shinmachi.fumie@nihon-u.ac.jp (F.S.)

**Keywords:** buckwheat, albumin, α-amylase inhibitor, diabetes, hyperglycaemia

## Abstract

Inhibiting starch hydrolysis into sugar could reduce postprandial blood glucose elevation and contribute to diabetes prevention. Here, both buckwheat and wheat albumin that inhibited mammalian α-amylase in vitro suppressed blood glucose level elevation after starch loading in vivo, but it had no effect after glucose loading. In contrast to the non-competitive inhibition of wheat α-amylase inhibitor, buckwheat albumin acted in a competitive manner. Although buckwheat α-amylase inhibitor was readily hydrolysed by digestive enzymes, the hydrolysate retained inhibitory activity. Together with its thermal stability, this suggests its potential use in functional foods that prevent diabetes.

## 1. Introduction

Diabetes mellitus (DM) has a major impact on health worldwide, with the number of patients estimated to be 422 million in 2014 according to the latest survey that was conducted by World Health Organization (WHO) [1] (p. 25). DM is called a “silent killer” because patients often experience no obvious symptoms until they suddenly develop lesions, such as retinopathy, nephropathy, neuropathy, and angiopathy [1] (p. 13). These complications sometimes lead to blindness, renal failure, and food ulcer, which seriously affect health-related quality of life of patients. One factor contributing to prevention of DM and its complications is controlling the elevation in postprandial blood glucose levels by consuming an appropriate diet. Ingestion of a substance that inhibits polysaccharide hydrolysis is an effective means to suppress the elevation of blood glucose levels [2]. Therefore, α-amylase inhibitor (α-AI) has been attracting attention for its potential to prevent and treat DM.

Cereals often contain a high concentration of α-AI albumin proteins in seeds to resist against animals, like insects [3], including well-studied examples in cereals, such as wheat (*Triticum aestivum* L.), rice (*Oryza sativa* L.), barley (*Hordeum vulgare* L.), rye (*Secale cereal* L.), maize (*Zea mays* L.), and kidney beans (*Phaseolus vulgaris* L.) [4,5,6,7,8,9,10,11,12]. In particular, α-AIs from wheat and kidney beans strongly inhibit mammalian α-amylases and delay the hydrolysis of starch to reducing sugars [13,14,15]. Wheat α-AI inhibits α-amylase from both insects and mammals, suppressing blood glucose elevation in rats, dogs, and humans [16,17,18]. In addition, wheat α-AI shows considerable resistance to digestion by pepsin and trypsin, as well as thermal stability [19], and is therefore expected to maintain its inhibitory activity even after sterilization processes. Wheat α-AI has already been used as a functional component to suppress the elevation of blood glucose level in Food for Specified Health Uses (FoSHU) in Japan [20]. On the other hand, rice α-AI has strong resistance to hydrolysis by digestive enzymes and inhibits α-amylase from insects, but does not inhibit that from mammals [21]. However, we have shown that rice α-AI suppresses blood glucose elevation even after glucose loading, indicating a starch-independent mechanism, such as adsorbing glucose in the small intestine [21]. Both wheat and rice α-AIs are water-soluble albumin proteins that are tasteless and odourless and can therefore be included in almost any kind of food. Because rice α-AI suppresses postprandial hyperglycaemia through a different mechanism than that of wheat α-AI, simultaneous intake of these α-AIs may show a synergistic effect in suppressing blood glucose elevation. Furthermore, the different characteristics of rice α-AI as compared with wheat α-AI motivated us to discover other cereal α-AI proteins for anti-hyperglycaemic applications.

Buckwheat (*Fagopyrum esculentum* Moench) also contains a proteinaceous α-AI albumin fraction that is known to inhibit porcine pancreatic α-amylase [22,23]. Buckwheat flour has been used as an ingredient mainly for noodles and pancakes in Asian and European countries. Since many people in these countries have experience of eating buckwheat, buckwheat α-AI is considered to be acceptable as a food additive for anti-hyperglycaemia. However, the detailed characteristics of buckwheat α-AI and its effect in vivo have not yet been investigated. Therefore, in this study, we examined the suppressive effect of buckwheat α-AI on postprandial hyperglycaemia and characterised its enzyme-inhibition mechanism, digestibility, and thermal stability, the latter being an important property in food processing.

## 2. Materials and Methods

### 2.1. Materials

Buckwheat flour (Tomizawa Shoten Co., Ltd., Tokyo, Japan), wheat flour (Nisshin Flour Milling Inc., Tokyo, Japan), and mealworms were purchased from a local market. α-Amylases from the human pancreas and saliva and porcine pancreas were obtained from Sigma-Aldrich (St. Louis, MO, USA), and 2-chloro-4-nitrophenyl-α-D-maltotrioside (G3-CNP), the substrate for α-amylase, was from Oriental Yeast (Tokyo, Japan). α-Amylase from mealworms was prepared, as described by Buonocore and Poerio, with some modifications [24]. Pepsin from porcine stomach mucosa and trypsin from bovine pancreas were obtained from Wako Pure Chemical Industries (Osaka, Japan). All other chemicals used were of reagent grade.

### 2.2. Preparation of Buckwheat and Wheat α-AIs

Buckwheat and wheat α-AIs were prepared according to the method that was described by Feng et al., with some modifications [7]. Buckwheat flour or wheat flour was mixed with 5 times its weight of 25 mM 4-(2-hydroxyethyl)piperazine-1-ethanesulfonic acid (HEPES) buffer at pH 6.9 for 3 h at 4 °C and then centrifuged at 15,000× *g* for 15 min at 4 °C. The supernatant was heated at 80 °C for 20 min to denature non-heat-stable proteins and centrifuged at 15,000× *g* for 15 min at 4 °C. The clear supernatant was subjected to ammonium sulphate fractionation; the protein fraction precipitating at 40% (NH_4_)_2_SO_4_ was collected by centrifugation at 15,000× *g* for 60 min at 4 °C. The precipitate was dialysed against distilled water to re-solubilise the protein and centrifuged at 15,000× *g* for 15 min at 4 °C. After lyophilisation of the supernatant, about 15 mg of protein dissolved in 20 mL of distilled water was applied to a Sephadex G-50 column (φ2.5 × 100 cm) (GE Healthcare UK Ltd., Buckinghamshire, UK) and equilibrated and eluted with distilled water at a flow rate of 0.2 mL/min, the absorbance of the eluate being measured continuously at 280 nm. Fractions (5 mL) were collected and the α-amylase inhibitory activity in each fraction was measured (see Section 2.3 below). The fractions showing more than 90% inhibitory activity against α-amylase from porcine pancreas were collected and lyophilised. α-AI powder was stored at −20 °C until use.

### 2.3. Measurement of α-Amylase Inhibitor Activity

The α-amylase inhibitory activity was measured, as described by Foo and Bais, with some modifications [25]. The inhibitory activity against α-amylase from mammals was determined by measuring the absorbance of 2-chloro-nitrophenol (CNP) at 405 nm produced from the cleavage of G3-CNP by α-amylase in the presence or absence of α-AI. The standard α-amylase inhibition assay was carried out by preincubating 25 μL of 1.6 U/μL α-amylase solution with 25 μL of 1 μg/μL cereal α-AI solution for 30 min at 37 °C in 20 mM HEPES buffer at pH 6.9 containing 50 mM NaCl and 3 mM CaCl_2_. The reaction was initiated by the addition of 50 μL of G3-CNP and incubated for 10 min at 37 °C. The enzyme reaction was terminated by the addition of 100 μL of 10% (*v*/*v*) Tris solution, after which the absorbance at 405 nm of the 2-chloro-4-nitrophenol that was produced by the reaction was measured. The control mixture was prepared by replacing the α-AI solution with 20 mM HEPES buffer at pH 6.9 containing 50 mM NaCl and 3 mM CaCl_2_. The inhibitory activity against α-amylase from mealworms was measured by the same procedure except that the buffer used was 20 mM acetate buffer at pH 5.4 containing 100 mM NaCl and the incubating temperature was 25 °C. The α-amylase inhibitory activity was expressed as percent inhibition relative to control using the following equation.

Inhibition percent (%) = (Ac − Ai)/Ac × 100(1)

where Ai and Ac are enzyme activities with and without an inhibitor, respectively.

### 2.4. Animals

Male Wistar rats seven weeks of age were purchased from Japan SLC (Shizuoka, Japan). The rats were acclimatised for a period of 7 days. Throughout the acclimatisation and subsequent study periods, rats were maintained in controlled environment of 23 ± 1 °C and 55% humidity under a 12-h light/dark cycle with light from 8:00 to 20:00. All rat experiments were performed in accordance with the Guidelines for Animal Experiments of the College of Bioresource Science of Nihon University (Approval numbers: AP11B012 and AP12B059).

### 2.5. Oral Starch and Glucose Tolerance Tests

The oral starch tolerance test (OSTT) and oral glucose tolerance test (OGTT) were conducted according to the methods described by Ina et al. [21] with some modifications. The OSTT and OGTT were carried out under non-anaesthesia conditions. After seven rats in each group were fasted overnight for 14 h, 300 mg/kg of buckwheat α-AI or wheat α-AI was orally administered as a mixture with phosphate-buffered saline containing 1 g/kg body weight soluble starch or glucose. Then, blood was taken from the tail vein at 0, 15, 30, 45, and 90 min. Blood glucose levels were measured with the Dexter-ZII meter (Bayer, Osaka, Japan) and plasma insulin was measured by ELISA (Rat Insulin ELISA Kit (U-E type); Shibayagi, Gunma, Japan). The area under the curve (AUC) was calculated for blood glucose and plasma insulin according to the methods that were described by Wolever and Jenkins [26].

### 2.6. Analysis of In Vitro Digestibility by Digestive Enzymes

Hydrolysates were prepared in vitro by pepsin and trypsin, as described by Ma and Xiong and Iwami et al., with some modifications [27,28]. First, 10 mg of α-AI suspended in 1 mL of 0.1 mg/mL pepsin in HCl adjusted to pH 2.0 was incubated at 37 °C for 2 h. After the pepsin was inactivated by neutralisation with 1 mL of 4% (*w*/*v*) NaHCO_3_, 1 mL of 1 mg/mL trypsin in 50 mM Tricine buffer at pH 8.0 was added and the mixture was incubated at 37 °C for 2, 4, or 6 h. The enzymatic hydrolysis was stopped by heating the sample solution at 100 °C for 5 min. The degree of hydrolysis was evaluated by the analysis of residual amylase inhibitory activity (see Section 2.3 above) and sodium dodecyl sulphate-polyacrylamide gel electrophoresis (SDS-PAGE). SDS-PAGE was carried out by the method of Laemmli [29]. After electrophoresis, the gels were stained for protein with 0.025% (*w*/*v*) Coomassie Brilliant Blue R-250 solution (Wako Pure Chemical Industries, Osaka, Japan).

### 2.7. Glycoprotein Staining

After SDS-PAGE, the fractionated protein was transferred electrophoretically to a polyvinylidene fluoride (PVDF) membrane (ProBlott, Applied Biosystem, Foster City, CA, USA). The membrane was washed three times with TPBS (phosphate-buffered saline containing 0.05% Tween 20) and immersed in a periodic-acid solution (TPBS containing 0.05% periodic acid). After washing three times with TPBS, the membrane was immersed in a biotin-hydrazide solution (25 µg/mL (+)-Biotin hydrazide (B7639, SIGMA-ALDRICH JAPAN, Tokyo, Japan)) dissolved in dimethyl sulfoxide. Then, the membrane was washed three times with TPBS and it was immersed in a horseradish peroxidase (HRP)-conjugated streptavidin solution (HRP-conjugated streptavidin (Funakoshi Co., Ltd., Tokyo, Japan) diluted with TPBS. The membrane was washed three times again with TPBS. To detect sugar chain bound to buckwheat α-AI, chemiluminescent reagent (Amersham™ ECL™ Western Blotting Analysis System, RPN2109, GE Healthcare UK Ltd., Buckinghamshire, UK) was used. The resulting light emission was detected using a gel imaging system (ChemiDoc MP, BioRad, Hercules, CA, USA).

### 2.8. Kinetic Analysis of α-Amylase Inhibition

The inhibitory activity of buckwheat α-AI against α-amylase from porcine pancreas was measured, as described by Seri et al. with some modifications [30] and compared with that of wheat α-AI. The α-amylase inhibitory activities of 0.05, 0.15, 0.25, and 0.35 mg/mL buckwheat and wheat α-AI were measured and the data were plotted according to the Lineweaver-Burk method [31].

### 2.9. Thermal Stability Analysis

The thermal stability of α-AI was evaluated by measuring the inhibitory activity against α-amylase from porcine pancreas after heating. α-AI was dissolved in 1 mL of distilled water to 0.1% (*w*/*w*) and boiled at 100 °C for 10, 30, 60, or 120 min. After cooling to room temperature, α-amylase inhibitory activity was measured as previously described in Section 2.3. The percent of unheated α-AI inhibitory activity remaining after heat treatment was defined as thermal stability, and was calculated as follows.

Thermal stability (%) = IAh/IAn × 100(2)

where IAh and IAn are α-amylase inhibitor activity of heated α-AI and that of non-heated α-AI, respectively.

### 2.10. Statistical Analysis

The data were represented as mean ± standard error (S.E.) The values were evaluated by one-way analysis of variance followed by the post-hoc Tukey-Kramer multiple range test.

## 3. Results

### 3.1. α-Amylase Inhibitory Activity

**Figure 1:**
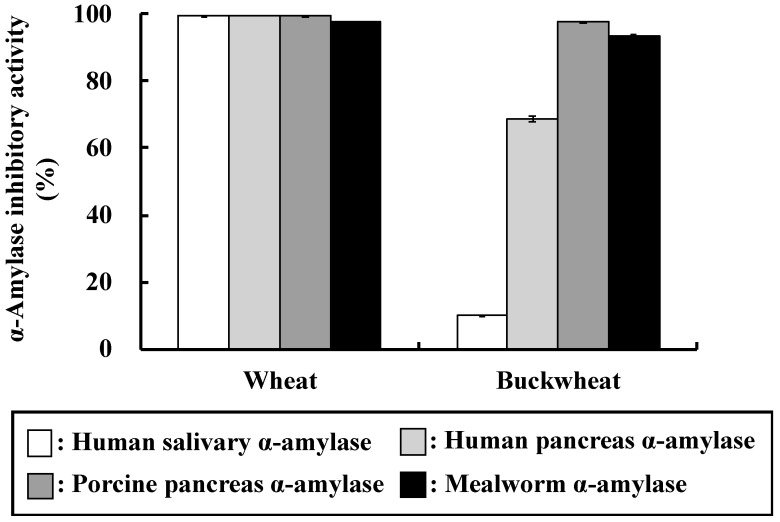
α-Amylase inhibitory activity of wheat and buckwheat α-AIs. Each value is the mean of three experiments with standard error (S.E.) shown as a vertical bar.

The inhibitory activity of buckwheat and wheat α-AI against α-amylase from several sources is shown in Figure 1. Wheat α-AI strongly inhibited α-amylase from human saliva (99.5%), human pancreas (99.3%), porcine pancreas (99.4%), and mealworm (97.6%). On the other hand, buckwheat α-AI also strongly inhibited α-amylase from porcine pancreas (97.9%) and mealworm (93.2%), but it showed somewhat decreased inhibition of α-amylase from human pancreas (68.7%) and only very weak inhibition of that from human saliva (10.2%).

### 3.2. Oral Starch and Glucose Tolerance Test

**Figure 2:**
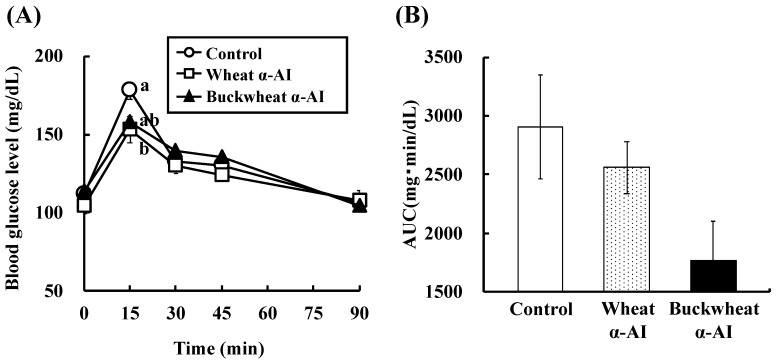
Effect of wheat and buckwheat α-amylase inhibitors (α-AIs) on (**A**) blood glucose level and (**B**) glucose area under the curve (AUC) after oral starch tolerance test using Wistar rats. Each value is the mean of 6–7 experiments with S.E. shown as a vertical bar. Values with different letters are significantly different at *p* < 0.05.

**Figure 3:**
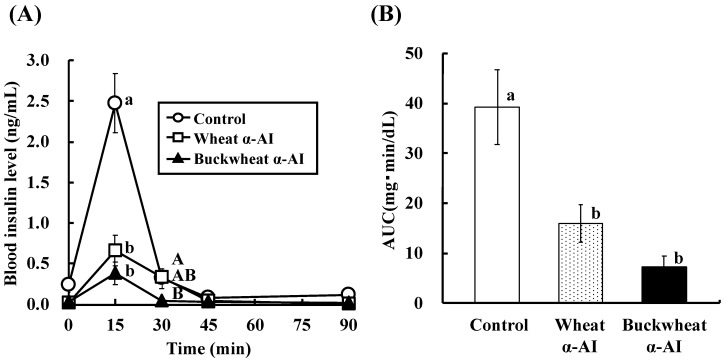
Effect of wheat and buckwheat α-AIs on (**A**) plasma insulin level and (**B**) insulin AUC after oral starch tolerance test using Wistar rats. Each value is the mean of seven experiments with S.E. shown as a vertical bar. Values with different letters are significantly different at *p* < 0.05.

**Figure 4:**
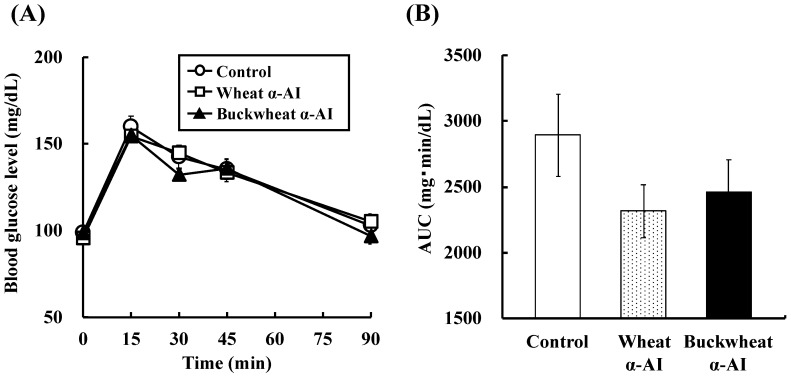
Effect of wheat and buckwheat α-AIs on (**A**) blood glucose level and (**B**) glucose AUC after oral glucose tolerance test using Wistar rats. Each value is the mean of seven experiments with S.E. shown as a vertical bar.

**Figure 5:**
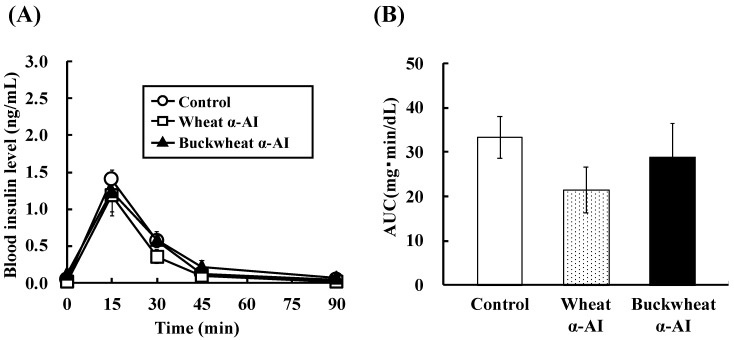
Effect of wheat and buckwheat α-AIs on (**A**) plasma insulin level and (**B**) insulin AUC after oral glucose tolerance test using Wistar rats. Each value is the mean of seven experiments with S.E. shown as a vertical bar.

The effect of buckwheat and wheat α-AIs on blood glucose and plasma insulin levels after starch loading was examined in normal rats. The postprandial blood glucose levels 15 min after starch loading of rats administered buckwheat and wheat α-AIs were 12% and 15% lower, respectively, than those of the rats used as a control group (Figure 2). At the same time point after starch loading, the postprandial plasma insulin levels of rats that were administered buckwheat and wheat α-AIs were 85% and 70% lower, respectively, than those of control rats (Figure 3). When the same experiment was conducted with glucose loading rather than starch loading, buckwheat, and wheat α-AIs did not suppress postprandial blood glucose elevation (Figure 4) or plasma insulin level (Figure 5).

### 3.3. In Vitro Digestibility and Glycoprotein Staining

**Figure 6:**
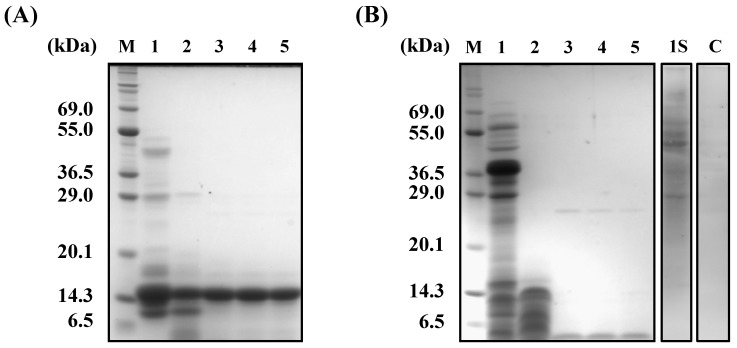
SDS-PAGE of wheat and buckwheat α-AIs before and after treatment with digestive enzymes and glycoprotein staining. (**A**) Wheat α-AI; (**B**) Buckwheat α-AI. (M) Marker; (1) Undigested; (2) Digested by pepsin for 2 h; (3) Digested by pepsin for 2 h followed by digestion with trypsin for 2 h; (4) Digested by pepsin for 2 h followed by digestion with trypsin for 4 h; and, (5) Digested by pepsin for 2 h followed by digestion with trypsin for 6 h; (1S) Undigested and stained with glycoprotein-staining reagent; (C) Bovine serum albumin stained with glycoprotein-staining reagent.

**Figure 7:**
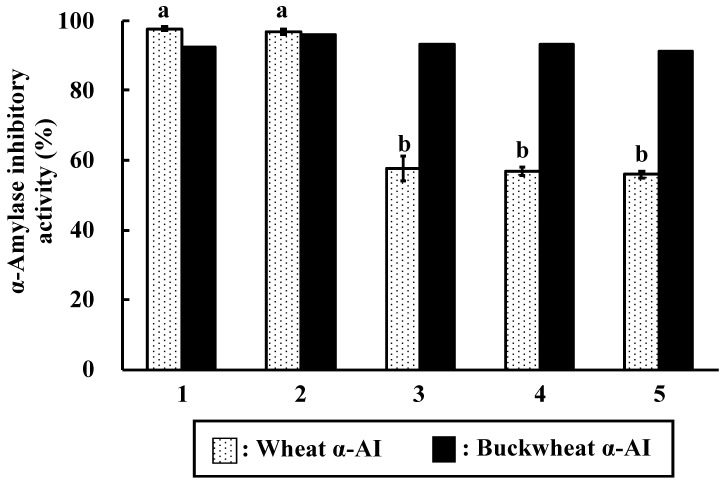
α-Amylase inhibitory activity of wheat and buckwheat α-AIs after digestion. (1) Undigested; (2) Digested by pepsin for 2 h; (3) Digested by pepsin for 2 h followed by digestion with trypsin for 2 h; (4) Digested by pepsin for 2 h followed by digestion with trypsin for 4 h; and, (5) Digested by pepsin for 2 h followed by digestion with trypsin for 6 h. Values with different letters are significantly different at *p* < 0.05.

The in vitro protein digestibility of α-AI was examined using sequential digestion by pepsin and trypsin. The 14-kDa wheat protein showed high resistance to digestion (Figure 6A), whereas the buckwheat protein was mostly hydrolysed to peptides that were smaller than 6.5 kDa, indicating that buckwheat α-AI is not resistant to digestive enzymes. The remaining α-amylase inhibitory activity of buckwheat and wheat α-AIs was examined after treatment by digestive enzymes (Figure 7). Although buckwheat α-AI was hydrolysed by digestive enzymes, it retained high inhibitory activity against α-amylase (91.4%). On the other hand, the α-amylase inhibitory activity of wheat α-AI decreased to 55.9% of its original level after treatment by pepsin and trypsin in spite of its resistance to digestion.

Buckwheat α-AI of higher molecular weight (> 29 kDa) was stained with glycoprotein-staining reagent. On the other hand, glycoprotein was hardly detected in bovine serum albumin, which was used as a negative control.

### 3.4. Kinetic Analysis of α-Amylase Inhibition

**Figure 8:**
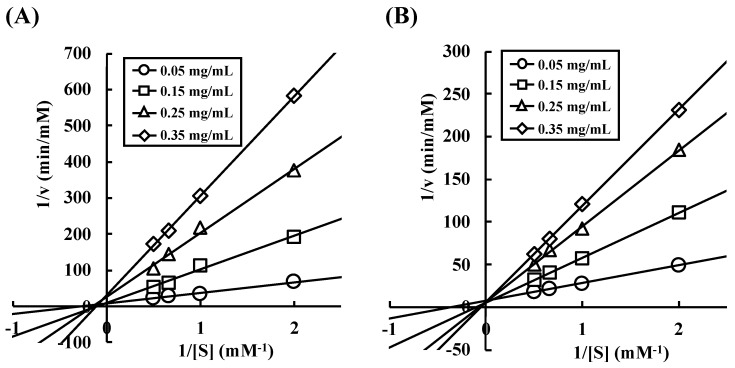
Kinetic analysis of the inhibitory activity against α-amylase from porcine pancreas. (**A**) Wheat α-AI; and, (**B**) Buckwheat α-AI.

Lineweaver-Burk plots were generated to assess the enzyme kinetics of wheat (Figure 8A) and buckwheat (Figure 8B) α-AIs. The plots of wheat α-AI intersected on the same abscissa section. On the other hand, the plots of buckwheat α-AI intersected on the same ordinate section.

### 3.5. Thermal Stability

**Figure 9:**
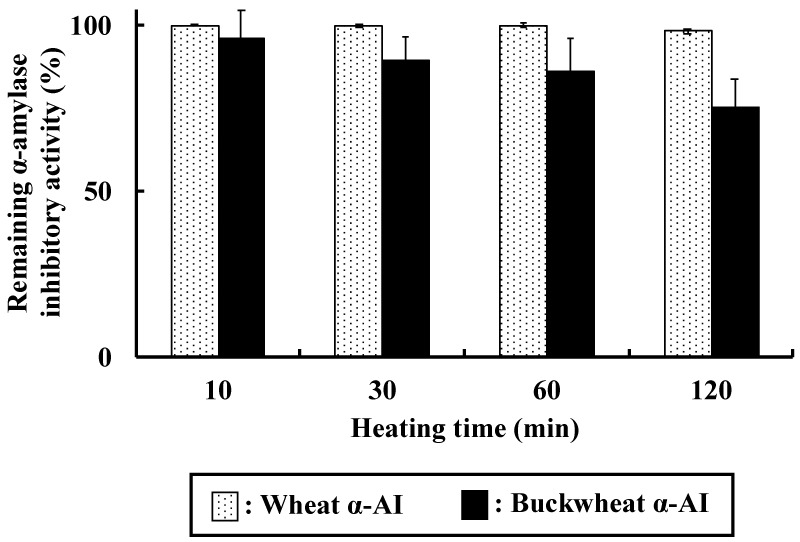
Thermal stability of wheat and buckwheat α-AIs. Each value is the mean of 2–3 experiments with S.E. shown as a vertical bar.

To characterise the heat stability of buckwheat and wheat α-AIs, the inhibitory activity against porcine pancreatic α-amylase was measured after heating at 100 °C for 10–120 min (Figure 9). Both wheat and buckwheat α-AIs maintained high inhibitory activity even after heating at 100 °C for 120 min (98.2% and 75.4%, respectively).

## 4. Discussion

This study demonstrated for the first time that buckwheat α-AI suppressed postprandial blood glucose elevation in rats. Wheat α-AI has already been reported to inhibit α-amylase from both insects and mammals [4,5,6,19], and it has a suppressive effect on blood glucose elevation [16,17,18]. As a result of its thermal stability [19], which allows it to retain activity even after sterilisation, wheat α-AI has already been used as a functional component in FoSHU in Japan [20]. In the present study, the α-amylase inhibitory activity and suppressive effect on postprandial hyperglycaemia of buckwheat α-AI were compared with those of wheat α-AI. In addition, the thermal stability of buckwheat α-AI was evaluated as a means to predict its stability during the sterilisation and cooking processes necessary to produce food products.

Various cereals contain α-AIs, some of which inhibit α-amylase from mammals and others that from insects only. As shown in Figure 1, wheat α-AI inhibited α-amylase from human saliva, human pancreas, porcine pancreas, and mealworms. We have reported that rice α-AI inhibits α-amylase from mealworms, but it does not inhibit α-amylase from human saliva, human pancreas, and porcine pancreas [21]. α-AI from barley, rye, maize, and kidney bean are also reported to inhibit α-amylase from mealworms [8,9,10,11,12]. On the other hand, buckwheat α-AI inhibited α-amylase from human pancreas, porcine pancreas, and mealworms, but did not inhibit that from human saliva (Figure 1), which is similar to the results of Feng et al., Ikeda et al., and Buonocore et al. [7,23,24]. These results imply that the mechanisms of substrate recognition of α-amylases from human saliva and pancreas are different and the structures of buckwheat and wheat α-AIs are not identical. Although the in vitro α-amylase inhibitory activity of buckwheat α-AI was about 30% less than that of wheat, these results encouraged us to investigate the suppressive effect of buckwheat α-AI on hyperglycaemia in vivo.

In OSTT, buckwheat α-AI suppressed the elevation in blood glucose and plasma insulin levels slightly more strongly than wheat α-AI (Figure 2 and Figure 3), while neither α-AI suppressed the elevation upon OGTT (Figure 4 and Figure 5). The results for wheat α-AI were similar to those of Puls and Keup [16]. We have reported that rice α-AI suppresses blood glucose elevation both on OSTT and OGTT. Because rice α-AI does not inhibit α-amylase from mammals but is not hydrolysed by digestive enzymes, its suppressive effect on blood glucose elevation after glucose loading is assumed to be due to the adsorption of glucose molecules onto its indigestible structure in the small intestine in a similar action as that of dietary fibre [21]. Because buckwheat α-AI inhibited α-amylase from mammals in vitro, as shown in Figure 1, its effect on postprandial hyperglycaemia after starch loading can likely be attributed to inhibiting the hydrolysis of starch to reducing sugars, similar to the mechanism of wheat α-AI.

Although the in vitro α-amylase inhibitory activity of buckwheat α-AI was less than that of wheat α-AI (Figure 1), the in vivo anti-hyperglycemic effect of buckwheat α-AI was higher than that of wheat α-AI (Figure 2 and Figure 3). To explain these contradictory phenomena, we evaluated the α-amylase inhibitory activity after digestion in vitro. The α-amylase inhibitory activity of wheat α-AI decreased to 60% after treatment with pepsin followed by trypsin (Figure 7), though the protein showed resistance to digestion (Figure 6). On the other hand, although buckwheat α-AI was hydrolysed to low-molecular-weight peptides by digestive enzymes (Figure 6), it retained almost 100% α-amylase inhibitory activity even after digestion (Figure 7). These results suggest that the wheat α-AI was partially digested in vivo, reducing the suppressive effect on hyperglycaemia, while the hydrolysate of buckwheat α-AI possessed high α-amylase inhibitory activity. This may explain why buckwheat α-AI showed a more potent suppressive effect on hyperglycaemia in spite of its weaker α-amylase inhibitory activity in vitro when compared with wheat α-AI.

There are two hypotheses that explain the phenomenon of buckwheat α-AI hydrolysate having α-amylase inhibitory activity: (1) a certain peptide sequence shows α-amylase inhibitory activity, or (2) sugar chain covalently bound to some peptide shows α-amylase inhibitory activity. To explore these two possibilities, we investigated the enzyme-inhibition mechanism of wheat and buckwheat α-AIs. In Figure 8, the Lineweaver-Burk plots of wheat α-AI intersected at the same point on the abscissa, indicating that wheat α-AI inhibited the activity of α-amylase from porcine pancreas in a non-competitive manner, as previously reported [32]. On the other hand, the plots of buckwheat α-AI intersected at the same point on the ordinate, indicating that buckwheat α-AI inhibited α-amylase activity in a competitive manner. Because sugars commonly fit the active site of α-amylase and peptides are unlikely to be recognised as a substrate, glycopeptides produced from buckwheat α-AI would be the competitive inhibitors. Some researchers reported glycoproteins that were obtained from plants inhibit α-amylase in a competitive manner [33,34,35]. In addition, glycoproteins larger than 29 kDa were detected in undigested buckwheat α-AI (Figure 6B). Considering that free sugars should have been removed during the preparation of buckwheat α-AI and most buckwheat albumin was hydrolysed to be peptides smaller than 6.5 kDa, glycopeptides that were produced by digestive enzymes from glycoproteins present in buckwheat α-AI might have exhibited α-amylase inhibitory activity. Therefore, as the mechanisms of α-amylase inhibitory activity of buckwheat and wheat α-AIs differed with each other, these would be proteins of different structure and molecular weight.

Both wheat and buckwheat α-AIs maintained high α-amylase inhibitory activity after heating, as shown in Figure 9. Oneda et al. reported that wheat α-AI showed high thermal stability [19], consistent with our results showing that it retained more than 98% of α-amylase inhibitory activity even after heating at 100 °C for 120 min, probably due to intramolecular disulphide bonds. The α-amylase inhibitory activity of buckwheat α-AI gradually decreased but it was still 75% after heating at 100 °C for 120 min. This result is consistent with our assumption that sugar chain in buckwheat α-AI shows α-amylase inhibitory activity and thus retained the activity, even after denaturation by heating. The thermal stability of buckwheat α-AI is high enough to be used in food products that undergo sterilisation.

## 5. Conclusions

In conclusion, buckwheat α-AI suppressed postprandial hyperglycaemia after starch loading by inhibiting α-amylase activity in a competitive manner. Buckwheat α-AI retained its inhibitory activity against α-amylase, even after digestion and heating. Therefore, it is a good candidate for use as a functional component in FoSHU, such as foods to suppress the elevation of blood glucose levels and prevent diabetes.
